# The molecular tumor board as a step in cancer patient management: a southern Italian experience

**DOI:** 10.3389/fmed.2024.1432628

**Published:** 2024-09-11

**Authors:** Stefania Tommasi, Leonarda Maurmo, Alessandro Rizzo, Claudia Carella, Girolamo Ranieri, Simona De Summa, Francesco Mannavola, Vincenzo Emanuele Chiurì, Michele Guida, Claudia Nisi, Michele Montrone, Francesco Giotta, Margherita Patruno, Rosanna Lacalamita, Brunella Pilato, Francesco Alfredo Zito, Livia Fucci, Claudio Antonio Coppola, Paolo Ditonno, Patrizia Nardulli, Davide Quaresmini, Sabino Strippoli

**Affiliations:** ^1^Unità di Diagnostica Molecolare e Farmacogenetica, IRCCS Istituto Tumori Giovanni Paolo II Bari, Bari, Italy; ^2^Unità Operativa di Oncologia Medica, IRCCS Istituto Tumori Giovanni Paolo II Bari, Bari, Italy; ^3^Unità di Oncologia Interventistica, IRCCS Istituto Tumori Giovanni Paolo II Bari, Bari, Italy; ^4^Unità di Oncologia Medica, Azienda Ospedaliera Policlinico Consorziale di Bari, Bari, Italy; ^5^Unità di Oncologia Medica, Ospedale "Sacro Cuore di Gesù" Gallipoli, Gallipoli, Italy; ^6^Reparto di Oncologia, Ospedale San Giuseppe Moscati Taranto, Taranto, Italy; ^7^Centro Studi Tumori eredo-familiari, IRCCS Istituto Tumori Giovanni Paolo II Bari, Bari, Italy; ^8^Unità Operativa di Anatomia Patologica, IRCCS Istituto Tumori Giovanni Paolo II Bari, Bari, Italy; ^9^Unità Operativa di Ematologia, IRCCS Istituto Tumori Giovanni Paolo II Bari, Bari, Italy; ^10^Unità Operativa Farmacia e U.M.A.C.A., IRCCS Istituto Tumori Giovanni Paolo II Bari, Bari, Italy

**Keywords:** Molecular Tumor Board, precision medicine, comprehensive genomic profile, team, liquid biopsy

## Abstract

**Introduction:**

The management of cancer patients follows a Diagnostic Therapeutic and Care Pathway (PDTA) approach, aimed at achieving the optimal balance between care and quality of life. To support this process, precision medicine and innovative technologies [e.g., next-generation sequencing (NGS)] allow rapid identification of genetic-molecular alterations useful for the design of PDTA-approved therapies. If the standard approach proves inadequate, the Molecular Tumor Board (MTB), a group comprising specialists from diverse disciplines, can step in to evaluate a broader molecular profile, proposing potential therapies beyond evidence levels I–II or considering enrolment in clinical trials. Our aim is to analyze the role of the MTB in the entire management of patients in our institute and its impact on the strategy of personalized medicine, particularly when all approved treatments have failed.

**Materials and methods:**

In alignment with European and national guidelines, a panel of clinicians and preclinical specialists from our institution was defined as the MTB core team. We designed and approved a procedure for the operation of this multidisciplinary group, which is the only one operating in the Puglia region.

**Results and discussion:**

In 29 months (2021–2023), we discussed and analyzed 93 patients. A total of 44% presented pathogenic alterations, of which 40.4% were potentially actionable. Only 11 patients were proposed for enrollment in clinical trials, treatment with off-label drugs, or AIFA (the Italian pharmaceutical agency for drugs)—5% funding. Our process indicators, time to analysis, and number of patient cases discussed are in line with the median data of other European institutions. Such findings underscore both the importance and usefulness of the integration of an MTB process into the care of oncology patients.

## 1 Introduction

Precision medicine is a healthcare approach that takes into account an individual’s unique characteristics, such as genetic profile, lifestyle, environment, and personal health history. It aims to provide more effective and targeted treatments while minimizing potential side effects and adverse reactions. The Diagnostic Therapeutic and Care Pathway (PDTA) is a clinical-care-organizational scheme that contemplates the discussion of each patient’s clinical history in a multidisciplinary team of pathology (MTPs) (1). This allows precision medicine to optimize effectiveness and efficiency within a specific local healthcare setting. In the Istituto di Ricovero e Cura a Carattere Scientifico (IRCCS) Istituto Tumori Giovanni Paolo II (ITGPII), regardless of the type of neoplasia, PDTA ensures streamlined access for patients through a centralized point of contact (Oncology Orientation Center: CoRO), along with a clearly defined and standardized treatment pathway. In our region, Puglia, prevalent cancers such as lung, breast, uterus, and prostate have regional PDTAs in place, seamlessly integrated into institutional frameworks focused on efficient patient management.

Currently, the approach to a patient’s neoplasia is completely changing from a histological to a mutational approach, which focuses on understanding the genetic mutations and alterations that occur in cancer cells (2). It involves the analysis of genomic data to identify specific mutations, genetic variations, and alterations in cancer cells and tumors. Advances in genomic technologies for sequencing profiling, such as NGS, have become more accessible and affordable, allowing the identification of disease-specific genetic changes that can serve as targets for tailored therapies (3). These techniques also consent to efficient and comprehensive analysis of cancer genomes, enabling researchers to identify specific mutations in cancer-associated genes and characterize the genomic landscape of tumors (4).

The PDTA therefore considers the possibility of analyzing mutational targets in accordance with Italian laws on the adequacy of the level of evidence (LoE) according to the European Society of Medical Oncology (ESMO) scale for clinical actionability of molecular targets (ESCATs) (5). However, the unavailability of further types of treatment can be overcome by a deep analysis of the molecular profile, looking for any actionable target.

The Molecular Tumor Board /MTB) is a group of professionals appointed to discuss the opportunity to perform an extended analysis and to discuss the results with the aim to recommend the most effective treatment options for patients to their clinicians (6).

The present paper describes the process followed at ITGPII in which we have integrated the MTB discussion into the standard approach to treatment and analyzed the results achieved, highlighting their impact on the therapeutic processes.

## 2 Materials and methods

The lean management approach is used in ITGPII to improve operational efficiency and eliminate non-value-added activities in patients’ care, as reported in the integrated plan of activity and organization 2023–2026 (7) focusing on the overall flow of patients, processes, information, and materials.

Each patient is received by a Unit called Co.R.O. and assigned to the specific MTP. The MTP, as defined by the European Partnership for Action Against Cancer (EPAAC), is “a coordinated group of all medical and health professions involved in a specific disease, whose therapeutic approach is guided by the will to make shared evidence-based clinical decisions and to coordinate the delivery of care at each stage of the therapeutic process, encouraging patients to be active participants in this care pathway” (8, 9).

Each MTP follows PDTAs or discusses cases in weekly meetings in a multidisciplinary context consisting of local oncologists, radiologists, pathologists, molecular biologists, surgeons, radiation oncologists, psychologists, and any additional specialists as required by the specific settings. If the patient has progressed without the possibility of further standard therapies and his performance status allows it, the MTP includes this patient in the MTB discussion. Occult primary tumors are also discussed in the MTB to look for both pathognomonic and druggable mutations.

**Figure 1:**
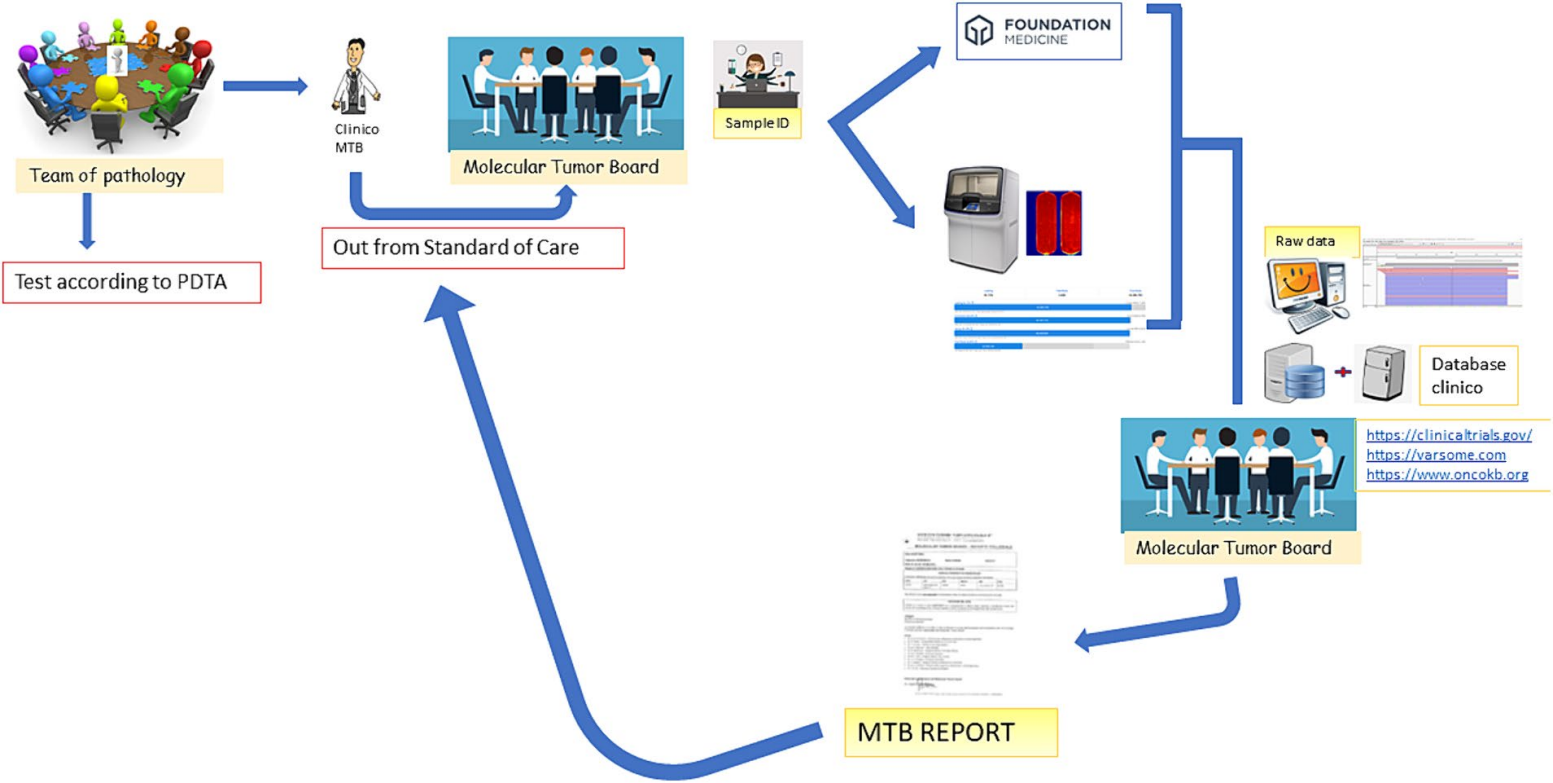
MTB workflow. The figure emphasizes that the patient is discussed in the MTB by a clinician after the decision of the Multidisciplinary Pathology Team (MTP). A data manager provides the patient ID and initiates the analysis *in-house* or by outsourcing. The molecular biologists perform the analysis and call up the variants annotated by the bioinformatician. All data are discussed again in the MTB and the possibility of treatment is discussed (off-label, Italian trials, AIFA funds, etc.). After discussion, the report is sent back to the clinician.

The MTB group consists of seven specialists, as shown in Table 1, who meet virtually every other week for at least 1 h. The methodological workflow of the MTB is summarized in Figure 1. The agenda for the day is sent to the participants by the data manager and includes molecular results of patients already analyzed and new patient data sheets to be discussed.

**Table 1:** MTB components of ITGPII with respect to Italian Medical Oncology Association (AIOM) guidelines.

	MTB ITGPII BARI	AIOM
Oncologists of different specialties	13	X
Anatomic-Pathologists	2	X
Biologists	2	X
Geneticist	1	X
Clinical pharmacologist		X
Bioinformatician	1	X
Pharmacologist	2	X
Research nurse		X
Data manager	1	
Bioethicist		X
Clinical epidemiologist		X
Patient association representative		X

While all patient clinical cases are discussed in MTP meetings, only a selection of them are discussed in MTB meetings. Enrolled patients must meet the following criteria: age ≥ 18 years; life expectancy ≥12 weeks; performance status ECOG ≤2; absence of significant comorbidities; adequate biohumoral indices; exhausted standard therapeutic lines according to the specific indications for each histology from the guidelines or PDTA; disease with resistance to available standard treatments; rare orphan diseases with no codified therapies; clinical and preclinical evidence suggesting possible therapeutic relevance of non-routinely evaluated targets; and incidental molecular target identified in previous analyses with evidence of treatment efficacy.

All patients signed informed consent for the collection, storage, and use of biological material and associated data to perform molecular investigations of potential therapeutic interest and, eventually, for research. The ethical committee approved this project, the information for patients, and the informed consent (protocol n. 1036/21).

The MTB decides the most appropriate type of analysis to perform, deciding between a short or extended profile. They also assess whether to analyze available tumor tissue or a liquid biopsy.

### 2.1 Molecular analysis

Patients for whom targets with a LoE I–II are relevant are routinely profiled using panels of approximately 20–50 genes and are not discussed in the MTB.

Only patients without actionable targets of LoE I–II are discussed in the MTB. These patients are profiled using panels of 50 genes or, more often, panels with >300 genes, depending on the pathology and the likelihood of highlighting targets for which off-label or ‘compassionate use’ drugs may be available. Molecular analysis is performed on tumor tissue or liquid biopsy (plasma) depending on the patient’s clinicopathological condition, lesions accessibility to biopsy, and MTB suggestion. Some analyses are led in-house using the NGS Thermo Fisher platforms and panels for the detection of mutations in 20–52 genes and specific fusion transcripts (we call it a short profile). In these cases, microsatellite instability (MSI) is also evaluated by real-time and fragment analyses. The turn-around time (TAT) of the “in-house” analysis is approximately 7–10 working days. When a larger number of genes needs to be investigated, including evaluating tumor mutational burden, the analysis is led in-house using the NGS Thermo Fisher platforms. However, it is more often outsourced to Foundation Medicine (we call it an extended profile). The timing in both these cases is approximately 10–15 working days.

In both in-house and outsourced results, all detected variants are first reviewed by a bioinformatics expert, a molecular biologist, and a medical geneticist to verify their pathogenicity, frame them in the ESCAT levels of evidence, and highlight any alterations to be studied at the germline level for suspected familial syndromes. In addition, all Italian clinical trials involving the use of the variant found are searched and suggested in the MTB discussion.

A consecutive series of 93 patients enrolled over 29 months from 2021 to 2023 were clustered according to the pathological alterations detected. Molecular clusters were designed using Oncoprint plots generated by the Complex Heatmap R package (9), incorporating both tissue and plasma analyses.

### 2.2 Process indicators

To monitor the performance of MTB, we have identified two process indicators: (1) TAT and (2) number of patients treated.

We defined TAT as the time from patient interview to molecular report. Considering the possibility that there are many different reasons why a patient cannot receive treatment, we reviewed each patient’s subsequent clinical history.

The laboratory has been ISO9001:2015 certified for diagnostic and research processes for 5 years and positively participates in European Molecular Quality Network (EMQN) EQA schemes.

## 3 Results

Since October 2020, the MTB has been organized at the ITGPII to allow people who have no further therapeutic chance, following PDTA, to highlight possible usable targets for the LoE Escat ≥3 therapies. Since our approach to patients follows a process that takes care of patients following the PDTAs, we used MTB as an area for improvement. First, a standardized procedure was implemented and legally approved regarding patient enrolment, supporting documentation for discussion, modality of analysis, potential treatment, and type of reporting.

**Figure 2:**
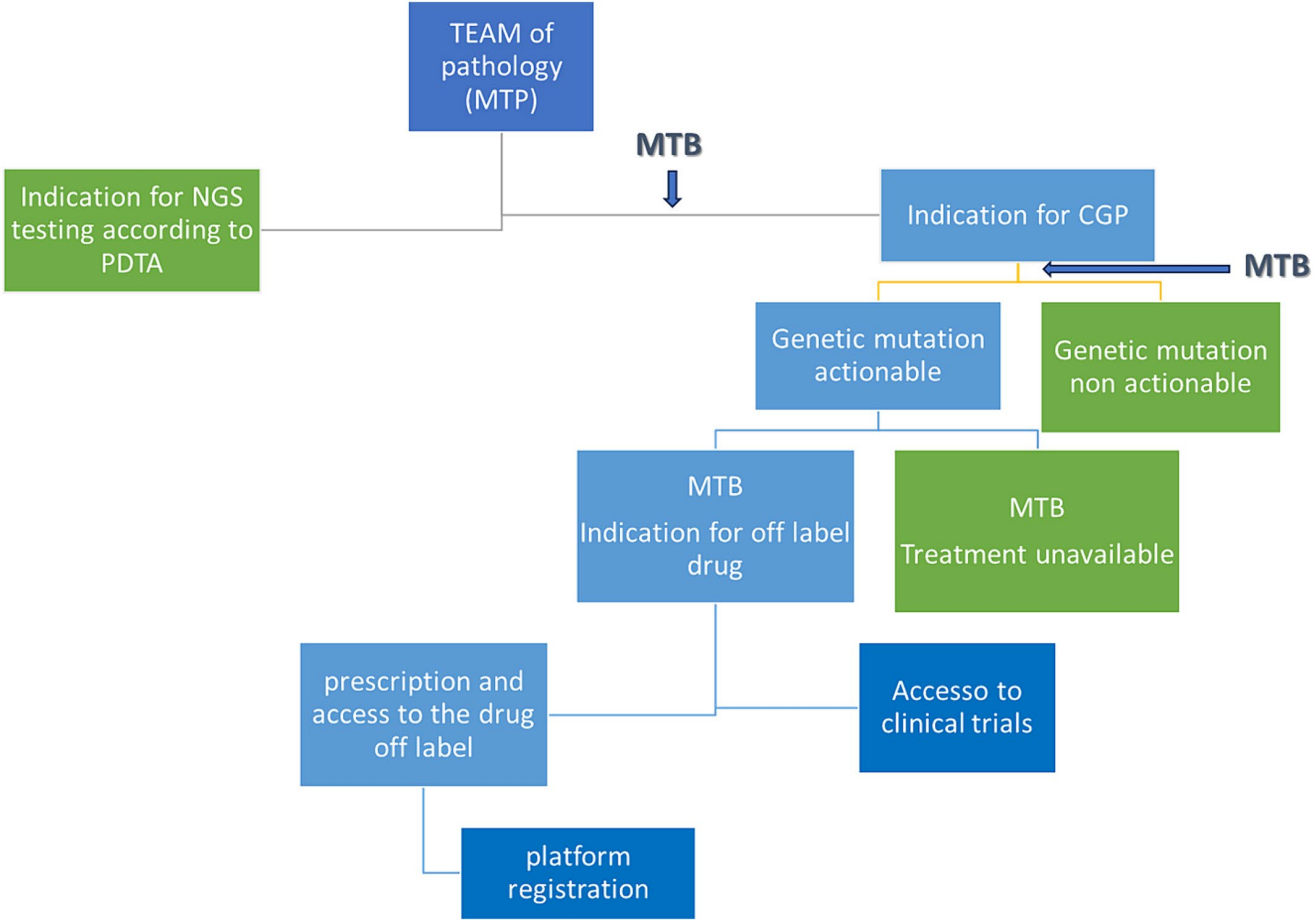
Decisional tree from patient discussion to eventual treatment. MTB, molecular tumor board; PDTA, diagnostic therapeutic and care pathway; MTP, multidisciplinary team of pathology; CGP, comprehensive genomic profile.

The decision algorithm is shown in Figure 2.

The inclusion and exclusion criteria for patients were decided in the MTB referring to the Italian Association for Medical Oncology (AIOM) recommendations^1^ and are highlighted in Table 2.

**Table 2:** Inclusion and exclusion criteria for MTB patient enrollment.

Inclusion criteria
Exhaustion of standard therapeutic lines in accordance with histology-specific indications from guidelines or PDTAs Cancer with resistance to available standard treatments Clinical and preclinical evidence of possible clinical and therapeutic relevance of targets not evaluated in routine Rare orphan diseases without coded therapies The presence of biomolecular data in previous analyses with the identification of molecular targets for which there is evidence of treatment Unusual clinical history based on which it is believed that performing extensive molecular profiling may have therapeutic implications Family history of inherited mutation (e.g., BRCA and MSH) to identify possible therapeutic targets and prognostic factors and to set prevention strategies for the patient and his family Age ≥ 18 years Life expectancy ≥12 weeks ECOG performance status ≤2 Signed informed consent for the collection, storage, and use of biological material and associated data for the purpose of performing biomolecular investigations for research and potential therapeutic interest Absence of significant comorbidities Adequate biohumoral indices

Exclusion criteria
Patients for whom access to therapies based on identified molecular targets is possible (unless they have shown resistance to such treatments) Patients eligible for active study protocols Patients eligible for nominal/expanded access protocols Patients with a life expectancy of less than 3 months Patients who have other therapeutic options are considered effective in treating the disease, except in cases with molecular alterations showing emerging evidence as resistance targets to available treatments Patients for whom it is not possible to obtain a tumor sample through tissue biopsy or liquid biopsy, suitable for molecular investigation

Figure 1 depicts the entire process: the clinician, following the decision of the Multidisciplinary Pathology Team MTP, presents the case of a patient to the MTB. A data manager provides the patient ID and initiates the analysis *in-house* or by outsourcing. The molecular biologists perform the analysis and call up the variants annotated by the bioinformatician. All data are discussed again in the MTB and the possibility of treatment is discussed (off-label, Italian trials, AIFA funds, etc.). After discussion, the report is sent back to the clinician.

**Figure 3:**
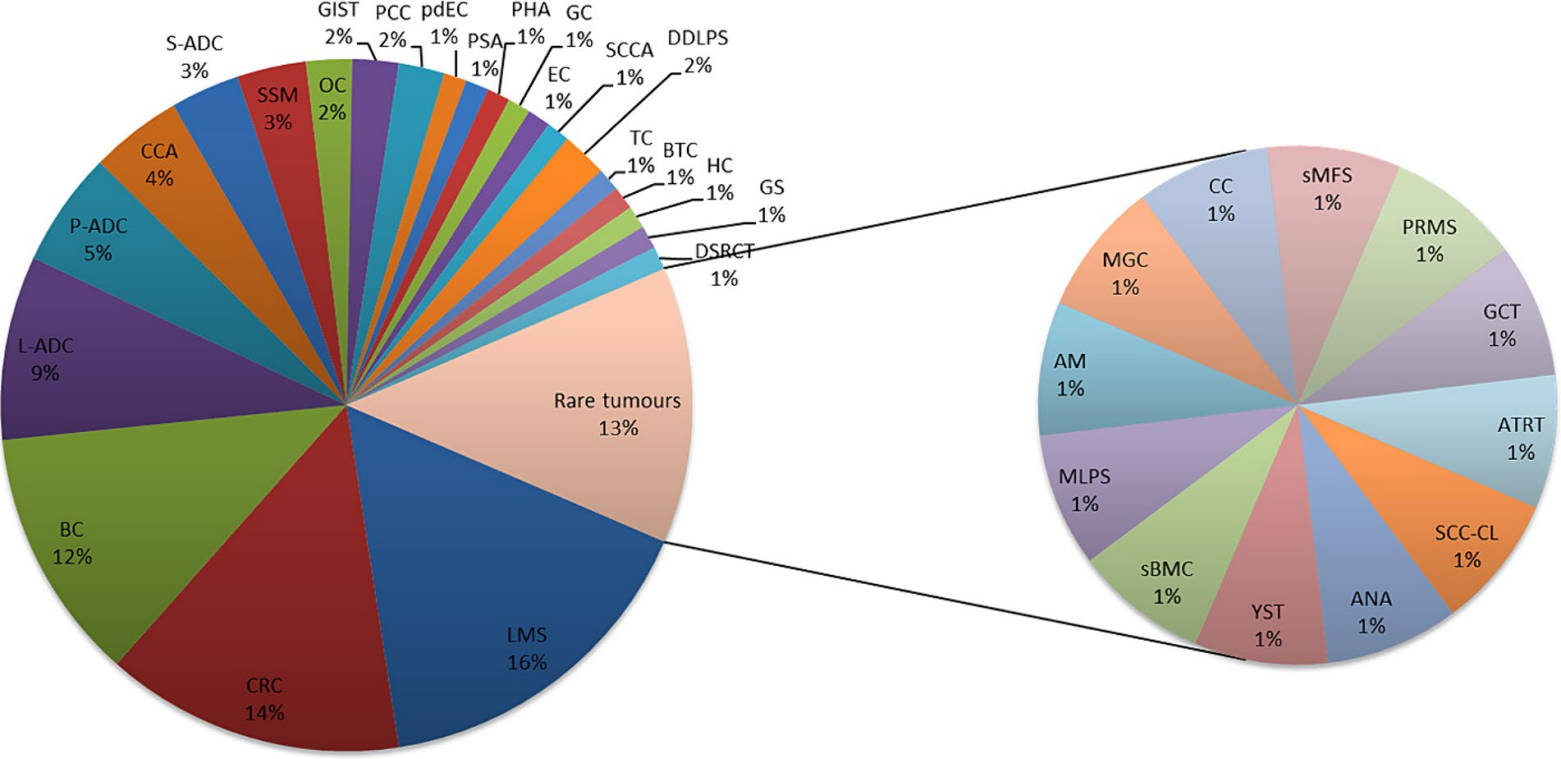
Pathologies discussed in MTB, are common and rare. Leiomyosarcoma (LMS); colon adenocarcinoma (CRC); breast carcinoma (BC); lung adenocarcinoma (L-ADC); pancreatic adenocarcinoma (P-ADC); cholangiocarcinoma (CCA); salivary adenocarcinoma (S-ADC); melanoma (SSM); ovarian carcinoma (OC); pheochromocytoma (PCC); poorly differentiated enteric-type carcinoma (pdEC); splenic angiosarcoma (PSA); angiosarcoma/hepatic carcinoma (PHA); gastric carcinoma (GC); endometrial carcinoma (EC); anal squamous cell carcinoma (SCCA); dedifferentiated liposarcoma (DDLPS); testicular carcinoma (TC); biliary tract carcinoma (BTC); hepatic carcinoma (HC); gliosarcoma (GS); desmoplastic small-round-cell carcinoma (DSRCT); squamous cell carcinoma in the cervical lymph nodes (SCC-CL); anaplastic astrocytoma (ANA); yolk sac tumor (YST); bone metastasis of carcinoma with sarcomatoid features (sBMC); myxoid liposarcoma (MLPS); atypical meningioma (AM); poorly differentiated mucinous carcinoma of gastrointestinal origin (MGC); clivus chordoma (CC); sarcoid myxofibrosarcoma (sMFS); pleomorphic rhabdomyosarcoma (PRMS); granulosa cell cancer (GCT); and atypical teratoid rhabdoid tumor (ATRT).

Ninety-three patients were discussed in the MTB from July 2021 to November 2023. The most common pathologies (Figure 3) were colon cancer (14%), breast cancer (11%), lung adenocarcinoma (9%), and pancreatic adenocarcinoma (5%), while 45.2% were rare tumors, diseases with a prevalence of fewer than six cases out of a population of 100,000 per year, as defined by the ESMO policy.^2^ All patients were in metastatic stage, have been treated with more than two lines of therapies, were in progression, and had no possibility of being further treated using standard AIFA-approved drugs.

### 3.1 Molecular profile

**Figure 4:**
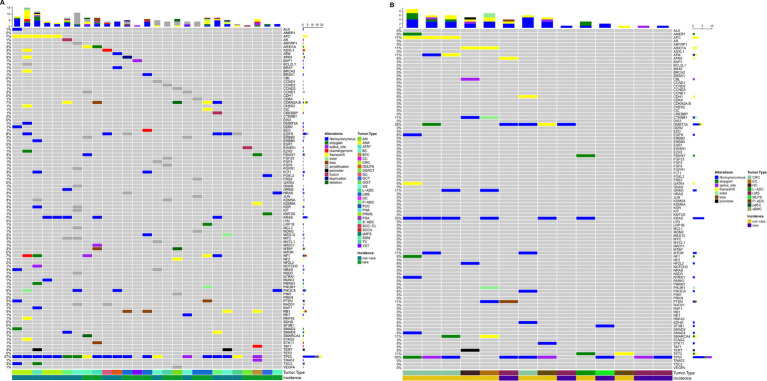
Oncoprint depicting alterations annotated in the tissue of patients where cases with no relevant alterations were excluded has been displayed **(A)**. Oncoprint depicting alterations annotated in the plasma of patients where cases with no relevant alterations were excluded has been displayed **(B)**. The numbers shown in the figure are the times when an alteration in a given gene was present.

As previously described, patients were profiled using panels of 50 genes or more often >300 genes depending on the pathology and the likelihood of highlighting targets for which some drugs can be administered off-label or according to ‘compassionate use.’ More than 250 pathogenic variants (*n* = 283) were detected, along with 642 unidentified variants (Figure 4). Only functionally relevant variants with allele frequencies >5% in tissue profiles and > 0.1% in liquid biopsy were considered. Pathogenic variants are detailed in Supplementary Table 1.

Forty-nine patients were studied by CGP, formalin-fixed paraffin-embedded (FFPE) tumor tissue was analyzed in 27 cases, and liquid biopsy was performed in 23 cases. Tissue from 4 patients resulted not evaluable: 2 for neoplastic cellularity <10% and 2 for low nucleic acid quality/quantity. Three patients with not evaluable tumor tissue underwent liquid biopsy analysis. One patient was profiled in both FFPE tissue and liquid biopsy, showing the same pathogenic alterations in both samples. One patient was profiled for a primary tumor and metastatic site, whose tissue was obtained shortly after the first consultation. The metastatic site lost the estrogen receptor 1 (ESR1) mutation but increased the TMB from 5 to 11 Mut/Mb.

Forty-four patients were profiled with a panel of 50 genes or were evaluated only by agnostic alterations (neurotrophic tyrosine receptor kinase (NTRK), MSI, etc.). Three patients were not evaluable due to low extracted nucleic acid quality/quantity.

Forty-six patients did not carry pathogenic alterations, regardless of rare disease status (Figure 4).

Nineteen percent (9/47) of the long-profiled patients had a tumor mutation burden >10mut/Mb, five in tissue analysis, and four in plasma. All patients analyzed for MSI were stable, but one.

### 3.2 Clinical evidence

Pathogenic variants were assessed for actionability using stratification according to the Clinical Actionability of Molecular Targets (ESCAT) classes and the LoE according to the Memorial Sloan Kettering (MSK) Cancer Center’s Precision Oncology Knowledge Base (5).

Most patients were discussed after more than three lines of therapy.

In 22 patients, the mutations detected were not actionable, and in 19 patients, the mutations were potentially actionable. Eight patients were suggested to be included in clinical trials: six in the Italian Rome protocol and two in other trials available in Italy. In two patients, literature evidence allowed to ask for the drug administered off-label. For 3 patients were suggested to acquire the specific drug according to AIFA Law 326/2003 art.48 (5% fund), while 6 patients there was no treatment available. Five patients were sent for genetic counseling.

Forty-five patients had passed away since the MTB discussion, while 24 are still alive with a median overall survival of 61.5 (0–114) months. Twenty-four patients were lost to follow-up.

### 3.3 Process results

In the 29 months of observation, our MTB has met 58 times. Some meetings have been avoided for lack of cases to be discussed, but some extraordinary MTB meetings have been organized to discuss results as soon as they were ready.

Time from discussion to MTB report has been counted: 18 working days needed for Comprehensive Genome Profile (CGP) analyses and 20 working days for short profile and singular analyses.

All patients who presented mutations that could be associated with a known syndrome with high allele frequency were recommended genetic counseling.

## 4 Discussion

In Italy, between 2007 and 2019, approximately 270,000 oncological deaths were avoided compared to those expected based on mortality rates from 2003 to 2006. Particularly, a 14.4% reduction in expected oncological deaths in men and a 6.1% reduction in expected oncological deaths in women (10). This is undoubtedly due to primary prevention, screening, and early diagnosis, but most importantly, innovative therapies and the ability to treat patients using genomic profiling according to the mutational approach (3). For this reason, the possibility of discussing peculiar cases in an MTB is becoming increasingly important.

We aim to demonstrate that it is possible to incorporate MTB into clinical practice as part of the improvement of the management of the oncology patient. At the moment, the MTB of our institute is the only MTB discussing patient cases in Puglia.

*A fortiori*, implementing an MTB prioritizes the focus on the patient. Following the results of more than 2 years of MTB discussion in our institute, MTB has been integrated into the management to complete patient care. Our results have shown that the establishment and implementation of MTB are possible and effective.

We identified as process indicators the time from the discussion of each case to the presentation of the report and the number of patients treated. In 29 months, we discussed the case of 93 patients who received a complete report in a mean time of 18 working days when a single run analysis was performed (CGP) and 20 days when separate analyses were needed: short gene panel (50 genes) plus MSI.

The interpretation of the MTB role and its modality of action are different among Italian regions (11): the criteria for case selection, the characteristics of the NGS panels to be used for molecular profiling, and the composition can be different across Italy. Thus, the number of patients enrolled per month ranged from 3 to 7 across different MTBs (12, 13), influenced by factors such as enrolment rules, the catchment area, wideness and compliance of oncologists to case discussions beyond the MTP. In other settings, such as Japan, a huge number of patients, up to 20 per month, are discussed due to the national health insurance system covering CGP testing (13) and easier drug accessibility rates.

The TAT to receive a report from an MTB, as reported in the literature, varies, ranging from 12.4 to 86 days (14, 15), thus our timing of 18–20 working days is reasonable to eventually treat patients.

In our experience, 6.4% of patients were ineligible due to a lack of tissue or analysis failure. The drawbacks associated with tumor biopsies are extensively documented (16): the process of retrieving archived samples frequently leads to notable delays, tissue is often insufficient, or the patient is unsuitable for biopsy. Almost 50% of patients presented pathogenetic alterations; however, only 40.4% of these presented potentially actionable mutations. This finding is notable considering the wide range reported in the literature, which spans from under 10% to approximately 90%. These disparities primarily stem from varying definitions of actionable alterations; furthermore, higher percentages of actionable alterations compared to earlier experiences in MTBs indicate the expanding landscape of available target therapy options (17). A clear comparison with literature data is not feasible as the percentage of druggable molecular alterations is conditioned by the types of histology included in the analysis as well as the types of analytical methodology used (comprehensive genomic profiling or NGS panel set for targetable mutations, as we did in 44% of cases). In this regard, it is noteworthy that in our series, 45% of the histology were sarcomas. The selection of this histology is related to the criteria chosen for MTB analysis, as they are characterized by poor outcomes and limited treatment options. The heterogeneity of sarcomas and the consequent lack of data on the incidence of druggable mutations in individual subtypes were further reasons for the prevalence of this histology in our series.

Although the percentage of targetable mutations reported in several case series (18, 19) on sarcomas varies considerably due to the influence of the percentage of different histotypes included, a common finding in our analysis is the prevalence of the TP53 mutation (20, 21). However, this mutation is not actionable in clinical practice, unlike the less common mutations of MDM2 and CDK4 found in our cases, whose value as predictive markers for response to specific drugs is being evaluated in a series of clinical trials that could change the therapeutic landscape in the treatment of sarcomas, which up to now has been dominated by a one-size-fits-all chemotherapy approach (22, 23).

Moreover, molecular analysis in some ultra-rare histotypes, such as gliosarcoma, has led to the detection of an ALK alteration in a single case—a finding that had not been previously reported. This alteration could represent a potential therapeutic target for a neoplasm currently lacking effective treatments.

For 42% of patients with actionable mutations, there was an indication for enrollment in clinical trials available in Italy. Very few patients would have been eligible for off-label drugs or drugs purchased with AIFA 5% funds (24).

MTB enables the customization of optimal oncological treatments for individual patients, addressing challenges posed by the interpretation of genomic alterations and the availability of targeted medications, while also facilitating access to clinical trials. The goal is to interpret complex molecular profiles and advise on a possible therapeutic approach, including the use of off-label drugs or referral of the patient to active clinical trials.

However, when clinical trials are unavailable, obtaining drugs through other described approaches can be very time-consuming. This delay represents a real limit, in the Italian scenario, affecting the efficiency and effectiveness of the MTB.

At the same time, in selected cases, MTB may have a key impact, as also reported in our practice. For example, among our included patients, a 49-year-old woman with metastatic lung cancer started treatment with Osimertinib following the results of the molecular analysis: EGFR duplication; after more than a year from the start of Osimertinib, the patient is still in treatment, and a partial response was observed. In another case, a 56-year-old patient with metastatic intrahepatic cholangiocarcinoma received first-line gemcitabine plus cisplatin. Following progressive disease and molecular testing according to the MTB, a BRAF V600E mutation was highlighted, and the patient started treatment with dabrafenib plus trametinib. Of note, the patient reported a progression-free survival of 7 months, with the partial response being the best response. In another case, a highly pretreated patient with salivary gland cancer received trastuzumab deruxtecan following molecular assessment ErbB2 amplification, as recommended by the MTB.

These examples further support the therapeutic opportunities offered by MTB and the importance of widespread adoption and use of MTB in Italy and abroad. In addition, a growing body of evidence worldwide has underscored this point, as well as the role of MTB in understanding the value of molecular alterations, interpreting test results, and assessing the benefit of targeted treatments. For example, a recently published study conducted at a tertiary center in India examined the proportion of patients who experienced a change in clinical management based on MTB recommendations, as well as the compliance of treating oncologists to these recommendations (25). Interestingly, the study conducted by Behel et al. reported that 60.7% of patients were recommended a change in clinical management, and compliance with the MTB recommendation to start a new systemic treatment was 60.5%.

In another recent multicenter study, El Helali et al. (26) investigated the feasibility of MTB in Hong Kong. A total of 122 cancer patients were enrolled, and 77 (63%) received treatment according to MTB recommendations. Notably, the authors observed that MTB-guided therapy could positively impact clinical outcomes. Based on these premises, and considering the growing interest in this important topic, MTBs should be considered fundamental elements of personalized cancer care, leading to the identification of treatment targets as well as the interpretation and discussion of therapies according to specific alterations, in order to provide personalized recommendations (27, 28).

## 5 Conclusion

Integrating the MTB as a decision-making moment in the PDTA of patients is possible and would allow precision medicine to be applied efficiently. However, to date, most patients with actionable alterations supported by evidence levels above II still face challenges accessing appropriate drugs. Certainly, the challenges related to timely drug access and biopsy limitations warrant further exploration. Furthermore, well-structured and well-designed, multicenter prospective studies are needed in this setting.

## Data Availability

The raw data supporting the conclusions of this article will be made available by the authors, without undue reservation.

## Footnotes

^1^https://www.aiom.it/wp-content/uploads/2020/11/2020_RaccTumorBoardMolecolare.pdf

^2^https://www.esmo.org/policy/rare-cancers-working-group/what-are-rare-cancers/definition-of-rare-cancers
