# Corrigendum: The molecular tumor board as a step in cancer patient management: a southern Italian experience

**DOI:** 10.3389/fmed.2024.1511339

**Published:** 2024-10-25

**Authors:** Stefania Tommasi, Leonarda Maurmo, Alessandro Rizzo, Claudia Carella, Girolamo Ranieri, Simona De Summa, Francesco Mannavola, Vincenzo Emanuele Chiurì, Michele Guida, Claudia Nisi, Michele Montrone, Francesco Giotta, Margherita Patruno, Rosanna Lacalamita, Brunella Pilato, Francesco Alfredo Zito, Livia Fucci, Claudio Antonio Coppola, Paolo Ditonno, Patrizia Nardulli, Davide Quaresmini, Sabino Strippoli

**Affiliations:** ^1^Unità di Diagnostica Molecolare e Farmacogenetica, IRCCS Istituto Tumori Giovanni Paolo II Bari, Bari, Italy; ^2^Unità Operativa di Oncologia Medica, IRCCS Istituto Tumori Giovanni Paolo II Bari, Bari, Italy; ^3^Unità di Oncologia Interventistica, IRCCS Istituto Tumori Giovanni Paolo II Bari, Bari, Italy; ^4^Unità di Oncologia Medica, Azienda Ospedaliera Policlinico Consorziale di Bari, Bari, Italy; ^5^Unità di Oncologia Medica, Ospedale “Sacro Cuore di Gesù” Gallipoli, Gallipoli, Italy; ^6^Reparto di Oncologia, Ospedale San Giuseppe Moscati Taranto, Taranto, Italy; ^7^Centro Studi Tumori eredo-familiari, IRCCS Istituto Tumori Giovanni Paolo II Bari, Bari, Italy; ^8^Unità Operativa di Anatomia Patologica, IRCCS Istituto Tumori Giovanni Paolo II Bari, Bari, Italy; ^9^Unità Operativa di Ematologia, IRCCS Istituto Tumori Giovanni Paolo II Bari, Bari, Italy; ^10^Unità Operativa Farmacia e U.M.A.C.A., IRCCS Istituto Tumori Giovanni Paolo II Bari, Bari, Italy

**Keywords:** Molecular Tumor Board, precision medicine, comprehensive genomic profile, team, liquid biopsy

In the published article, there was an error in the Funding statement. The Ministry of Health funding information is incomplete. It originally stated: “The author(s) that declare financial support was received for the research, authorship, and/or publication of this article. This research has been supported by 5×1000 2021 funds and the Ministry of Health 2023–2024 project funds.” The correct Funding statement appears below.

